# Parental smoking exposure before and during pregnancy and offspring attention-deficit/hyperactivity disorder risk: A Chinese child and adolescent cohort study

**DOI:** 10.3389/fpubh.2022.1017046

**Published:** 2022-10-10

**Authors:** Dong Liu, Yaping Ren, Tianfeng Wu, Huiping Shen, Peijing Yan, Yu Meng, Qianlong Zhang, Jun Zhang, Pinqing Bai, Jian Zhao

**Affiliations:** ^1^The Ministry of Education and Shanghai Key Laboratory of Children's Environmental Health, Xinhua Hospital, Shanghai Jiao Tong University School of Medicine, Shanghai, China; ^2^Department of School Health, Food Nutrition and Safety, Pudong New Area Center for Disease Control and Prevention, Shanghai, China; ^3^West China School of Public Health, Sichuan University, Chengdu, China

**Keywords:** attention-deficit/hyperactivity disorder (ADHD), paternal smoking, second-hand smoke (SHS) exposure, pregnancy smoking cessation, parental smoking

## Abstract

**Background:**

Previous studies revealed that maternal smoking exposure during pregnancy was an essential risk factor for offspring developing attention-deficit/hyperactivity disorder (ADHD). The impact of paternal smoking exposure 1 year before pregnancy on offspring ADHD risk is still unclear.

**Methods:**

The present study included 2,477 school-age children and their parents from the Shanghai Child and Adolescent Health Cohort who had complete data for offspring ADHD diagnosis and parents' smoking exposure before and during pregnancy information. A multivariate logistic regression model and Firth's logistic regression model were used to determine the associations of paternal smoking and parental smoke exposure patterns before and during pregnancy with offspring ADHD risk.

**Results:**

Children whose fathers smoked before pregnancy had a higher risk of developing ADHD [odds ratio (OR) = 2.59, 95% confidence interval (CI): 1.35–4.98] compared to those whose fathers had never been exposed to smoking. Similarly, parents who were exposed to smoking or second-hand smoke before pregnancy had 1.96 times (OR = 1.96, 95% CI: 1.19–3.22) more likely to have offspring with ADHD. Moreover, children whose parents were exposed to smoking both before and during pregnancy were 2.01 times (OR = 2.01, 95% CI: 1.29–3.12) more likely to develop ADHD.

**Conclusion:**

Paternal smoking before pregnancy and parental smoking exposure 1 year ahead of and throughout pregnancy were all risk factors for offspring developing ADHD.

## Introduction

Attention-deficit/hyperactivity disorder (ADHD) is a common chronic neuropsychiatric disorder affecting children worldwide (1). In China, approximately 6.25% of children and adolescents have ADHD (2). Compared to families with normal children, parents of children with ADHD suffer higher levels of stress and work-family conflict (3). An overall direct financial burden for a family reached 15,036 US dollars per child, and the annual costs of illness for children with ADHD were 124.5 billion US dollars (4). Thus, it is vital to identify the high-risk population and provide health intervention at an earlier stage such as before or during pregnancy.

Motivated by the developmental origins of health and disease (DOHaD) hypothesis, that offspring health outcomes might be caused by maternal exposure around pregnancy, researchers used to pay more attention to the impact of maternal exposures on offspring health outcomes than paternal exposures (5). Previous evidence revealed that parental smoking or exposure to nicotine during pregnancy were the risk factors for offspring developing ADHD (6–8). Recently, researchers found that the association of maternal smoking with offspring ADHD risk was stronger than that of paternal smoking (9). Compared with the global average smoking prevalence (33.5% for males and 6.7% for females) (10), there was a much higher prevalence of smoking among males (47.2%) and a lower prevalence among females (2.7%) in China (11). Moreover, approximately 83% of Chinese female smokers quit smoking during pregnancy (12). A recent study found that maternal SHS exposure during pregnancy was associated with offspring developing ADHD among Chinese children (13). However, data and evidence on the extent to which paternal smoking exposure 1 year before and during pregnancy affects offspring ADHD risk in the Chinese population are sparse. Controversially, a recent study found that maternal second-hand smoke (SHS) during pregnancy and maternal quitting smoking before pregnancy were not associated with offspring ADHD risk (14). Therefore, whether parents quitting smoking before pregnancy was associated with offspring ADHD risk, or how parents' smoking exposure patterns before and during pregnancy could influence the risk of developing ADHD among Chinese children remains unknown.

In this study, we examined the associations of paternal smoking and parental smoking exposure patterns before and during pregnancy with offspring ADHD risk using the data from the Shanghai Child and Adolescent Health Cohort (SCAHC).

## Methods

### Study design, participants, and data collection

The SCAHC is a prospective cohort study conducted by Shanghai Pudong New Area Centers for Disease Control and Prevention (CDC) since September 2020. SCAHC recruited 2,744 first-grade students with an average age of 7 years from 13 randomly selected elementary schools in the Pudong New Area, Shanghai, China, with a planned follow-up of 9 years until high school entry.

The baseline information was collected between November 2020 and December 2020. Parents were asked to complete baseline questionnaires including children's information, parents' information, children's lifestyle questionnaire, and children's sleep questionnaire. A total of 2,496 children completed the physical examination and biological specimen collection (blood, urine, and stool), and 2,549 families returned the questionnaires. After excluding those with missing/invalid values, data on 2,477 children and their parents were included in our analysis.

Written informed consent for using data collected via questionnaires, physical examinations, and biological samples was obtained from the participants. Ethical approval for this study was obtained from the Pudong New Area CDC/School of Public Health Fudan University Ethics Committee (2022-TYSQ-03-151).

### Outcome

A child who had been diagnosed with ADHD by a certified doctor in a secondary hospital (e.g., district-level hospital) or a higher-level hospital was defined as having ADHD in the present study. The diagnosis information was collected from parents via a questionnaire.

### Exposures

In this study, we used the phrase “before pregnancy” to represent “within 1 year before pregnancy” for simplicity. Paternal smoking status information before/during pregnancy was collected through two questions in the parents' self-reported questionnaire “Whether you smoked 1 year before the pregnancy (at least one cigarette per week for at least 1 year)?” and “Whether you smoked during the pregnancy (at least one cigarette per week for at least 1 year)?”. Fathers who answered “never” were defined as no (i.e., never smoked before or during pregnancy). All the others were defined as yes (i.e., smoked before or during pregnancy). Following the same classifications, due to few numbers of maternal smoking cases in our study (11 out of 2,477 mothers smoked before pregnancy and only one mother smoked during pregnancy), we used maternal SHS exposure before and during pregnancy as an alternative exposure. Maternal SHS exposure before/during pregnancy was collected through two questions “How many days per week you had been exposed to SHS 1 year before the pregnancy?” and “How many days per week you had been exposed to SHS during the pregnancy?”. Mothers who answered “never” were defined as no (i.e., not exposed to SHS before or during pregnancy), and others were defined as yes (i.e., exposed to SHS before or during pregnancy).

To assess the association between parental smoking exposure before/during pregnancy and offspring ADHD risk, the parental smoking exposure before pregnancy was categorized into four groups: (1) within 1 year before this pregnancy, fathers and mothers never smoked and been exposed to SHS, (2) maternal SHS exposure only, (3) paternal smoking only, and (4) both. Similar, parental smoking exposure during pregnancy was categorized into four groups: (1) during pregnancy, fathers and mothers never smoked and been exposed to SHS, (2) maternal SHS exposure only, (3) paternal smoking only, and (4) both paternal smoking and maternal SHS exposure.

**Figure 1:**
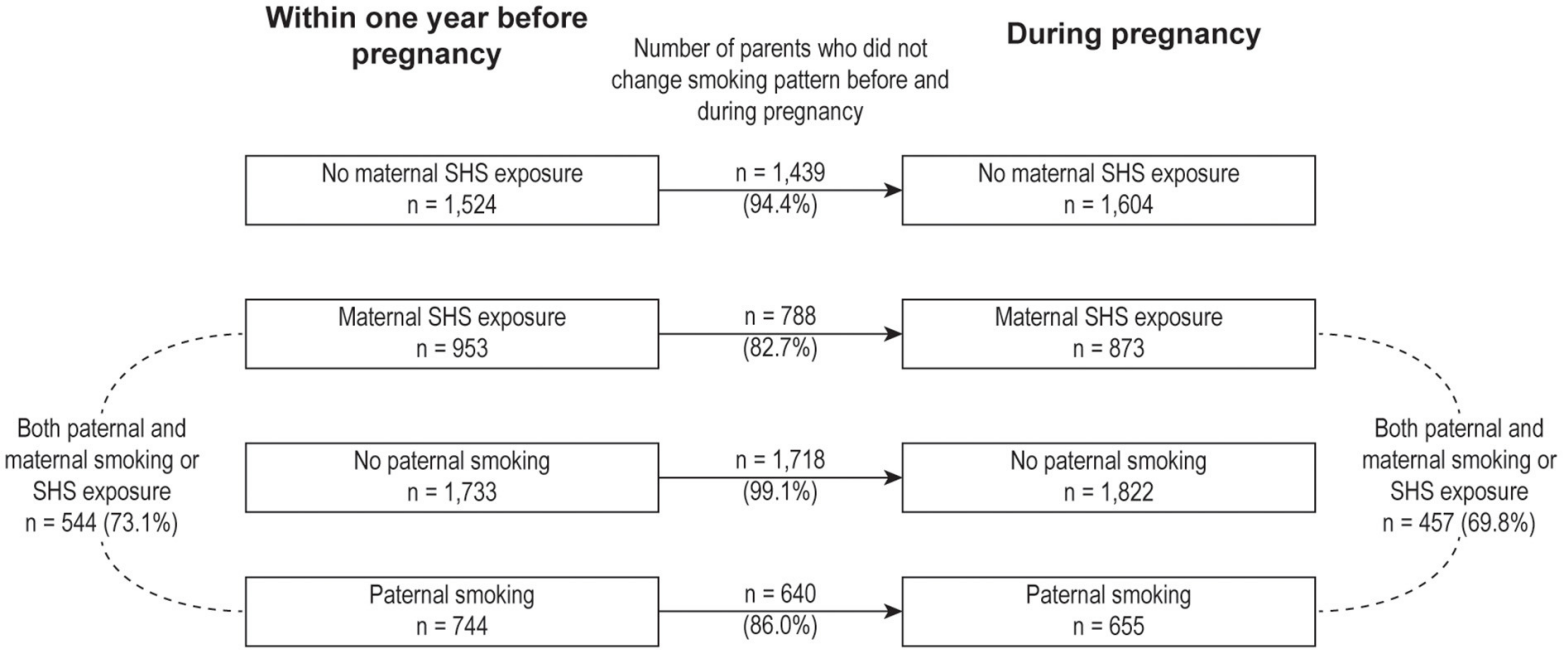
Parental smoking exposure pattern before and during pregnancy. The black lines represent the number of fathers or mothers who kept the same smoking or second-hand smoke exposure (or did not be exposed to smoking) patterns before and during pregnancy. The black dashed lines represent the number of both parents who were exposed to smoking or second-hand smoke. SHS, second-hand smoke.

**Figure 2:**
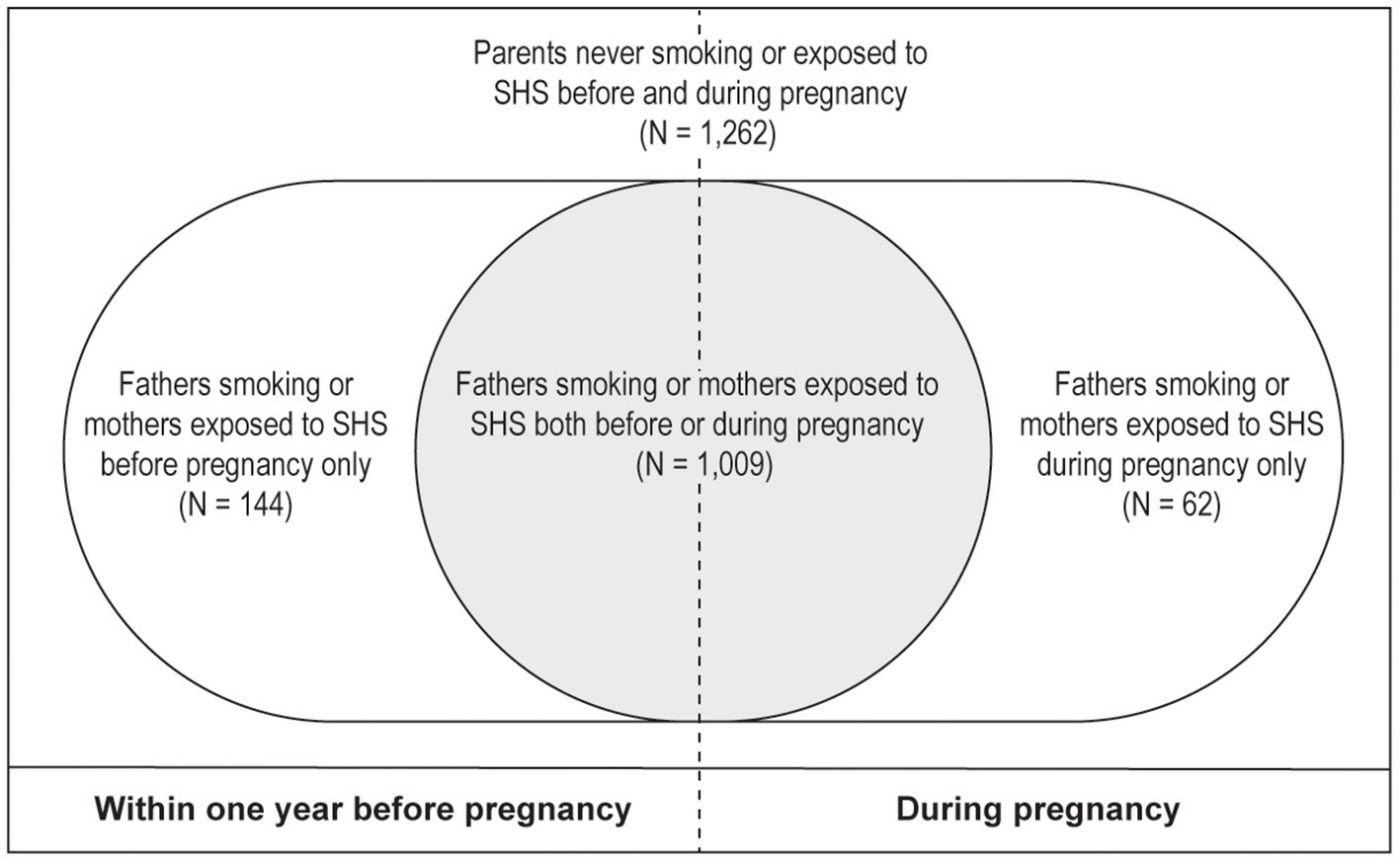
The number of parents of different smoking exposure patterns before and during pregnancy.

Given that over 80% of parents did not change their smoking or SHS exposure patterns during pregnancy (Figure 1). To further evaluate the associations between different parental smoking exposure patterns before/during pregnancy and offspring ADHD risk, we stratified parental smoking exposure patterns into four groups based on parental smoking exposure before/during pregnancy: (1) “Never been exposed to smoking” representing for those parents never been exposed to smoking before and during pregnancy, (2) “Parental smoking exposure before pregnancy only” indicated that fathers or mothers were exposed to smoking before pregnancy but never exposed to smoking during pregnancy (i.e., quitting smoking before pregnancy), (3) “Parental smoking exposure during pregnancy only” indicating that fathers or mothers been exposed to smoking during pregnancy but never been exposed to smoking before pregnancy, and (4) “Both” referring to that fathers or mothers were exposed to smoking before and during pregnancy (Figure 2).

### Potential confounders

Potential confounders were considered given the prior knowledge on common causes of parental smoking exposure and offspring ADHD risk (9, 15, 16). The confounders selected in this study included maternal alcohol consumption before pregnancy (yes or no), maternal alcohol consumption during pregnancy (yes or no), offspring gender (male or female), maternal age, maternal depression status during pregnancy (yes or no), paternal obesity [yes: body mass index (BMI) ≥ 25 kg/m^2^ or no: BMI < 25 kg/m^2^], and monthly income per capita (low: <6,000 Chinese Yuan (CNY) per month, middle: 6,000–10,000 CNY per month, or high: >10,000 CNY per month).

### Statistical analysis

Baseline characteristics of offspring and their parents were compared between offspring diagnosed with ADHD and offspring without ADHD using a *t*-test for continuous variables or a Chi-square test for categorical variables. A multivariate logistic regression model was used to examine the associations of parental smoking exposures on offspring ADHD risk with adjusting for potential confounders, including offspring gender, maternal alcohol consumption, maternal age, maternal depression status, paternal obesity, and monthly income per capita. Since there were few cases of paternal smoking and maternal SHS exposure before pregnancy or during pregnancy only, we used Firth's logistic regression to minimize the risk of bias suffering from a small number of cases (17). Chi-square test, *t*-test, and multivariate logistic regression were performed with R version 4.0.2 (18), and Firth's logistic regression was performed with SAS version 9.4 (19). A *P*-value < 0.05 was considered statistically significant.

## Results

A total of 2,477 children and their parents were included in the analysis of this study. The prevalence of ADHD diagnosed for children in this study was 4.2% (Table 1). The average maternal age at birth of the child for females who had a child diagnosed with ADHD was 27.1 years, which was 0.9 years younger than those without an ADHD offspring (*P* = 0.03). Among children with ADHD, 7.0% of fathers smoked before pregnancy and 7.6% of fathers smoked during pregnancy. Other baseline characteristics which were significantly associated with offspring ADHD risk include parents who were exposed to smoking both before and during pregnancy, offspring gender is male, maternal alcohol consumption before and during pregnancy, maternal depression during pregnancy, paternal obesity, and monthly income per capita lower than 6,000 CNY.

**Table 1:** The baseline characteristics of offspring and their parents participated in this study.

**Variables**	**Offspring diagnosed with ADHD**	**Offspring without ADHD**	***P*-value**
	**Mean (SD)**	**Mean (SD)**	
Maternal age	27.1 (4.1)	28.0 (4.1)	0.03
	***N*** **(%)**	***N*** **(%)**	
Sample size	103 (4.2%)	2,374 (95.8%)	
Parental smoking exposure before pregnancy			<0.001
Never	35 (2.6%)	1,289 (97.4%)	
Maternal only (SHS)	18 (4.4%)	391 (95.6%)	
Paternal only	14 (7.0%)	186 (93.0%)	
Both	36 (6.6%)	508 (93.4%)	
Parental smoking exposure during pregnancy			<0.001
Never	39 (2.8%)	1,367 (97.2%)	
Maternal only (SHS)	16 (3.8%)	400 (96.2%)	
Paternal only	15 (7.6%)	183 (92.4%)	
Both	33 (7.2%)	424 (92.8%)	
Parental smoking exposure pattern			<0.001
Never been exposed to smoking	32 (2.5%)	1,230 (97.5%)	
Parental smoking exposure before pregnancy only	7 (4.9%)	137 (95.1%)	
Parental smoking exposure during pregnancy only	3 (4.8%)	59 (95.2%)	
Both	61 (6.0%)	948 (94.0%)	
Maternal alcohol consumption before pregnancy			0.01
No	93 (3.9%)	2,269 (96.1%)	
Yes	10 (8.7%)	105 (91.3%)	
Maternal alcohol consumption during pregnancy			0.01
No	100 (4.1%)	2,366 (95.9%)	
Yes	3 (27.3%)	8 (72.7%)	
Offspring gender			<0.001
Females	25 (2.2%)	1,114 (97.8%)	
Males	78 (5.8%)	1,260 (94.2%)	
Maternal depression status during pregnancy			<0.001
No	71 (3.3%)	2,079 (96.7%)	
Yes	32 (9.8%)	295 (90.2%)	
Paternal obesity			0.002
No	50 (3.2%)	1,510 (96.8%)	
Yes	53 (5.8%)	864 (94.2%)	
Monthly income per capita (CNY)			0.003
Low (<6,000)	51 (5.7%)	845 (94.3%)	
Middle (6,000–10,000)	29 (4.4%)	634 (95.6%)	
High (>10,000)	23 (2.5%)	895 (97.5%)	

SHS, second-hand smoke; CNY, Chinese yuan; SD, standard deviation.

### Parental smoking exposure before and during pregnancy and offspring ADHD risk

The associations between parental smoking exposure and offspring ADHD risk are presented in Table 2, adjusting for maternal alcohol consumption, offspring gender, maternal age, maternal depression status during pregnancy, paternal obesity, and monthly income per capita. Children whose fathers were exposed to smoking within 1 year before pregnancy, but mothers were not exposed to SHS, were 2.59 times [odds ratio (OR) = 2.59, 95% confidence interval (CI): 1.35–4.98] more likely to develop ADHD compared to those whose parents had never been exposed to smoking or SHS. Similarly, during the pregnancy, parents that only fathers were exposed to smoking were 2.55 times (OR = 2.55, 95% CI: 1.36–4.79) more likely to have offspring with ADHD compared to non-smoking exposure parents. Meanwhile, children whose parents smoked and were exposed to SHS before or during pregnancy had a higher risk of developing ADHD (OR = 1.96, 95% CI: 1.19–3.22 or OR = 2.09, 95% CI: 1.27–3.44, respectively) compared to others whose parents had never been exposed to smoking or SHS.

**Table 2:** The associations of parental smoking exposure before/during pregnancy and offspring ADHD risk.

**Variables**	**Before pregnancy**		**During pregnancy**	
	**OR (95% CI)**	**Adjusted OR^a^ (95% CI)**	**OR (95% CI)**	**Adjusted OR^a^ (95% CI)**
**Parental smoking exposure**
Never	Ref	Ref	Ref	Ref
Maternal only (SHS)	1.70 (0.95, 3.03)	1.51 (0.83, 2.73)	1.40 (0.78, 2.54)	1.12 (0.61, 2.08)
Paternal only	2.77 (1.46, 5.25)	2.59 (1.35, 4.98)	2.87 (1.55, 5.31)	2.55 (1.36, 4.79)
Both^b^	2.61 (1.62, 4.20)	1.96 (1.19, 3.22)	2.73 (1.69, 4.39)	2.09 (1.27, 3.44)

OR, odds ratio; CI, confidence interval; Ref, reference group; SHS, second-hand smoke.

^a^Adjusted for maternal alcohol consumption, offspring gender, maternal age, maternal depression status during pregnancy, paternal obesity, and monthly income per capita.

^b^Paternal smoking and maternal second-hand smoke exposure before/during pregnancy.

### Parental smoking exposure patterns before and during pregnancy and offspring ADHD risk

The associations between parental smoking exposure pattern differences before/during pregnancy and offspring ADHD risk are presented in Table 3. Children whose parents were exposed to smoking both before and during pregnancy were 2.01 times (OR = 2.01, 95% CI: 1.29–3.12) more likely to develop ADHD compared with those whose parents had never been exposed to smoking after adjusting for potential confounders. There were no associations between parental smoking exposure before/during pregnancy only and offspring ADHD risk, and the adjusted ORs were 2.00 (OR = 2.00, 95% CI: 0.88–4.55) and 1.60 (OR = 1.60, 95% CI: 0.49–5.18), respectively.

**Table 3:** The associations of different parental smoking exposure patterns before and during pregnancy and offspring ADHD risk.

**Variables**	**OR (95% CI)**	**Adjusted OR^a^ (95% CI)**
**Parental smoking exposure pattern**
Never been exposed to smoking	Ref	Ref
Parental smoking exposure before pregnancy only	2.07 (0.91, 4.67)	2.00 (0.88, 4.55)
Parental smoking exposure during pregnancy only	2.23 (0.71, 6.97)	1.60 (0.49, 5.18)
Both^b^	2.46 (1.59, 3.79)	2.01 (1.29, 3.12)

OR, odds ratio; CI, confidence interval; Ref, reference group.

^a^Adjusted for sex of offspring gender, maternal age, maternal depression status during pregnancy, paternal obesity, and monthly income per capita.

^b^Parental smoking and second-hand smoke exposure both before and during pregnancy.

## Discussion

In this study, we found the associations between paternal smoking exposure before/during pregnancy and offspring ADHD risk. Children whose fathers smoked before pregnancy or children whose parents were exposed to smoking before and during pregnancy were more likely to develop ADHD during childhood than those whose fathers or both parents had never been exposed to smoking. Our findings are supported by previous studies which reported that parental smoking during pregnancy increased the risk for offspring developing ADHD (8, 14, 15, 20). Interestingly, due to a high correlation between paternal smoking and maternal SHS exposure in our study, we found that maternal SHS exposure during pregnancy was not associated with offspring ADHD risk after adjusting for paternal smoking.

Researchers found that smoking could affect the quality of semen and sperm (e.g., decreased semen volume, decreased sperm concentration and motility, deoxyribonucleic acid (DNA) strand breaks, DNA mutations, or higher DNA fragmentation), which could induce offspring at risk of adverse outcomes (21–26). A previous animal experiment showed that male mice exposed to nicotine could produce descendants with behavioral impairment (27). Notably, increased levels of reactive oxygen species (28, 29) and decreased levels of antioxidants (30), which affected semen quality and induced sperm DNA damage (31, 32), were observed in the seminal plasma of smokers.

Furthermore, paternal sex hormone levels change might play potential mediating roles between paternal smoking and sperm quality. Recent studies observed inverse associations between luteinizing hormone (LH) and sperm motility or morphology (26, 33), and elevated serum levels of LH and sex hormone-binding globulin were observed in male smokers more than in male nonsmokers (34–37). In addition, accumulating evidence supported that smoking has an impact on sperm DNA methylation (38, 39). Taken together, paternal smoking before pregnancy might affect sperm quality and could be a potential risk factor for offspring developing ADHD.

Meanwhile, the present results revealed that children whose parents smoked from 1 year before pregnancy to the end of pregnancy had a higher risk of developing ADHD than those whose parents never smoked. As per one of the referees' kind suggestions, we used parents who were exposed to smoking both before and during pregnancy as the reference group, the analysis results supported that parents who quit smoking before pregnancy (i.e., parental smoking exposure before pregnancy only) did not have a lower risk of having offspring with ADHD (OR = 1.00, 95% CI: 0.45–2.20), which was consistent with findings from a recent meta-analysis that smoking cessation before pregnancy did not associate with reduced offspring ADHD risk (14). In the present study, it suggested that children developing ADHD might be influenced by parental smoking exposure, mainly paternal smoking, before pregnancy. Therefore, future larger and prospective studies with a focus on the impact of smoking cessation time points on offspring ADHD risk are warranted.

Our study has a number of strengths. First, this cohort study has a large sample size in the urban area of China. Second, children's ADHD diagnosis information was obtained via the hospital which could minimize misclassification bias. Third, few maternal smoking cases and a high prevalence of paternal smoking provide a unique chance to assess the association between paternal smoking and offspring ADHD risk. Lastly, a high response rate of baseline recruitment (over 90%) could minimize potential selection bias.

There are several limitations to this study. First, data on parents' childhood ADHD diagnosis information were not available in this study due to lacking ADHD diagnostic standards in China when these parents were children decades ago. For this reason, we checked parents' family mental illness history, and we found that this variable did not confound the association between parental smoking and offspring ADHD risk in our study. Second, the pregnancy exposure information was self-reported by parents retrospectively. Therefore, recall bias is inevitable, though efforts such as public health professionals designing all the questionnaires and well-trained field workers conducting data collection, data cleaning, and quality control were made to minimize the recall bias. Third, detailed parental smoking information during different periods of pregnancy was not available.

## Conclusion

This study found that offspring were at an increased risk for developing ADHD when their fathers smoked within 1 year before pregnancy. Moreover, parental exposure to smoking from 1 year before pregnancy up until birth was a risk factor for offspring developing ADHD.

## Data availability statement

The raw data supporting the conclusions of this article will be made available by the authors, without undue reservation.

## Ethics statement

The studies involving human participants were reviewed and approved by Pudong New Area CDC/School of Public Health Fudan University Ethics Committee. Written informed consent to participate in this study was provided by the participants' legal guardian/next of kin.

## Author contributions

DL, YR, TW, PB, and JZhao: conceived this study. DL, YR, and YM: performed the data analysis. DL, YR, and JZhao: drafted the manuscript. HS, PY, YM, QZ, JZhan, PB, and JZhao: provided critical revisions. All the authors revised and approved the final version of the manuscript for submission.

## Funding

This study was funded by Pudong New Area Centers for Disease Control and Prevention, Shanghai, China (PKJ2021-Y29, PW2021A-01). This study was also partly funded by Xinhua Hospital, Shanghai Jiao Tong University School of Medicine, Shanghai, China (2021YJRC02).

## Conflict of interest

The authors declare that the research was conducted in the absence of any commercial or financial relationships that could be construed as a potential conflict of interest.

## Publisher's note

All claims expressed in this article are solely those of the authors and do not necessarily represent those of their affiliated organizations, or those of the publisher, the editors and the reviewers. Any product that may be evaluated in this article, or claim that may be made by its manufacturer, is not guaranteed or endorsed by the publisher.
